# Highly Localized Enrichment of *Trypanosoma brucei* Parasites Using Dielectrophoresis

**DOI:** 10.3390/mi11060625

**Published:** 2020-06-26

**Authors:** Devin Keck, Callie Stuart, Josie Duncan, Emily Gullette, Rodrigo Martinez-Duarte

**Affiliations:** Multiscale Manufacturing Laboratory, Department of Mechanical Engineering, Clemson University, Clemson, SC 29634, USA; dkeck@g.clemson.edu (D.K.); cdstuar@g.clemson.edu (C.S.); josied@g.clemson.edu (J.D.); egullet@g.clemson.edu (E.G.)

**Keywords:** sleeping sickness, Human African trypanosomiasis, trypanosoma, titanium, dielectrophoresis

## Abstract

Human African trypanosomiasis (HAT), also known as sleeping sickness, is a vector-borne neglected tropical disease endemic to rural sub-Saharan Africa. Current methods of early detection in the affected rural communities generally begin with general screening using the card agglutination test for trypanosomiasis (CATT), a serological test. However, the gold standard for confirmation of trypanosomiasis remains the direct observation of the causative parasite, *Trypanosoma brucei*. Here, we present the use of dielectrophoresis (DEP) to enrich *T. brucei* parasites in specific locations to facilitate their identification in a future diagnostic assay. DEP refers to physical movement that can be selectively induced on the parasites when exposing them to electric field gradients of specific magnitude, phase and frequency. The long-term goal of our work is to use DEP to selectively trap and enrich *T. brucei* in specific locations while eluting all other cells in a sample. This would allow for a diagnostic test that enables the user to characterize the presence of parasites in specific locations determined *a priori* instead of relying on scanning a sample. In the work presented here, we report the characterization of the conditions that lead to high enrichment, 780% in 50 s, of the parasite in specific locations using an array of titanium microelectrodes.

## 1. Introduction

Human African trypanosomiasis (HAT), also known as sleeping sickness, is a vector-borne neglected tropical disease endemic to rural sub-Saharan Africa. The disease is caused by infection of the protozoan *Trypanosoma brucei*, which is transmitted by the tsetse fly. Early diagnosis of the presence of *T. brucei* at the first stage of infection can have a significant impact on patient outcome by enabling timely and adequate treatment before the disease moves into a second stage. This is important because at this later stage the parasite penetrates the central nervous system, which leads to neuropsychiatric manifestations. such as sleep disorders, derangement or deep sensory disturbances that severely compromise the quality of life of the patient [1,2]. This second stage is fatal if untreated and drugs used to treat it are expensive and/or highly toxic. In contrast, drug therapy for early-stage HAT is effective and only mildly toxic [1,2]. 

Current methods of early detection in the affected rural communities generally begin with general screening using the card agglutination test for trypanosomiasis (CATT), a serological test. However, the gold standard for confirmation of trypanosomiasis remains the direct observation of the parasite [3]. Therefore, positive CATT readings are subsequently followed up through the direct observation of trypanosomes in blood, lymph node aspirates or cerebrospinal fluid (of note, examination of the cerebrospinal fluid after lumbar puncture is required to differentiate between HAT stages). In all cases, enrichment of the parasite in specific locations is crucial to facilitating their identification and different methods have been used to this end. 

The mini hematocrit centrifugation technique (mHCT), the quantitative buffy coat (QBC) and the miniature anion-exchange centrifugation technique (mAECT) are all techniques that use centrifugation, but for two different purposes. Centrifugal fractionation is used in mHCT and QBC to enrich the parasite by exploiting their difference in density with respect to blood cells. A hematocrit centrifuge is first used to fraction the blood sample (~50 µL of finger-prick blood) into plasma, buffy coat and red blood cell (RBC) layers in a capillary tube. The capillary tubes are then placed in a special holder and examined under a microscope by a trained eye to scan for the presence of parasites [4,5]. If present, they are expected to be concentrated near the interface between the buffy coat and the plasma layers. The difference between mHCT and QBC is that acridine orange, a fluorescent stain, and UV light, are used in QBC to facilitate parasite identification. However, both techniques can suffer from low specificity since enrichment is done only based on particle density. Contamination of the enriched sample with white blood cells (WBC) of similar density significantly complicates the identification of the parasite. In contrast, mAECT utilizes surface charge to enrich the parasite from the sample. At pH 6–9 *T. brucei* have been shown to have a neutral charge or be less negatively charged than blood cells. While centrifugation is also used in mAECT, this is done for the sole purpose of flowing the sample through a positively-charged column. Hence, the vast majority of the blood cells are retained in the column while the parasites are eluted and retrieved after the column. The use of mAECT has been shown to increase sensitivity over mHCT by 30% to 40% and significantly less contamination of the sample with other cells facilitates direct observation and identification of the parasite [6,7,8]. However, scanning the eluted sample for parasites is still required, and there remains the possibility that parasites are physically trapped in the column. 

Here, we present results on the use of dielectrophoresis (DEP) to enrich *T. brucei* parasites in specific locations to facilitate their identification in a diagnostic assay. DEP refers to physical movement that can be selectively induced on the parasites when exposing them to electric field gradients of specific magnitude, phase and frequency. This occurs thanks to the interaction between the electric field gradient and the electrical dipole induced on the parasite when this is exposed to such a field gradient. DEP can be generally classified as conventional or traveling-wave DEP [9,10,11,12,13,14], where conventional refers to short-distance movement and traveling wave to long-distance; and as positive DEP, occurring when targeted species move towards the electric field gradient, or negative DEP, when the targeted species move away from the gradient. The long-term goal of our work is to use conventional positive DEP to selectively trap and enrich *T. brucei* in specific locations while eluting all other cells in the sample. This would allow for a user to characterize the presence of parasites in specific locations determined *a priori* instead of relying on scanning a sample. In the work presented here, we report the characterization of the conditions that lead to high enrichment of the parasite in specific locations. 

DEP methods are advantageous over density-based techniques, such as centrifugation, due to their increase in specificity. In fact, different DEP signatures have been reported for blood cells and parasites [12,15,16]. Of most relevance to the work presented here is the separation of *T. brucei* from RBCs demonstrated by Kremer et al. using a light-induced DEP (LiDEP) setup [17], and the localization of *T. brucei* reported by Menachery et al. using spiral gold electrodes and traveling-wave DEP (twDEP) [18]. As previously detailed by one of us, there are different techniques to implement the field gradient required for DEP [19]. For instance, LiDEP relies on a light modulator; usually, a digital micromirror device and digital light processing technology coupled to optimized optics; and a photoconductive substrate to generate said gradient. Although Kremer et al. developed a portable LiDEP setup that was demonstrated for the manipulation of *T. brucei*, the complexity and cost of the instrumentation might not yield a practical application in the affected zones. TwDEP relies on a mobile or traveling electric field gradient, implemented through polarizing an electrode array with an AC field of alternating phases, and offers the ability for long-distance cell transportation through the interaction between the moving field and the polarized cell [20]. For example, twDEP could be desirable to move the *T. brucei* away from the original sample in an attempt to eliminate any background noise from remaining cells, or could eliminate the need for external forces for cell transport, i.e., centrifugation or micropumps. However, twDEP requires more sophisticated electronics than conventional DEP or LiDEP to apply AC fields of alternating phases. Furthermore, the low transport velocity achievable in twDEP may compromise the practicality of using such a technique in a diagnostic assay [13,18]. 

Building upon existent work on the use of DEP for *T. brucei* enrichment, we demonstrate the use of titanium microelectrodes to induce conventional DEP on *T. brucei* towards its enrichment in specific locations. Ti is a low-cost alternative to gold and other more expensive metals, which also offers biocompatibility and desirable mechanical properties for microfluidic devices [21,22,23,24]. The device presented here uses titanium planar microelectrodes that are patterned on a silicon wafer using batch processes standard in microfabrication. We expect that the relatively low cost of Ti and the straightforward fabrication approach will lead to inexpensive devices and a practical diagnostic assay in the future. 

## 2. Materials and Experimental Methods

### 2.1. Fabrication of the Experimental Device

**Figure 1:**
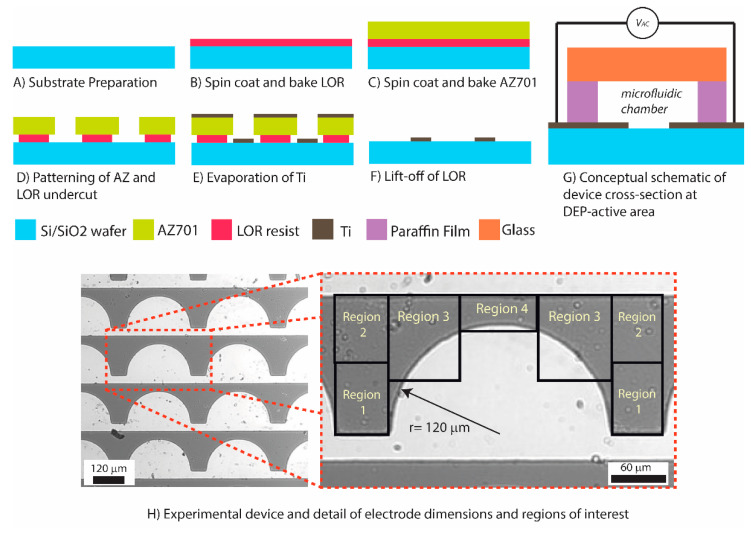
Fabrication of Ti electrodes: (**A**) A six-inch silicon substrate was descummed with oxygen plasma treatment at 20 uTorr. (**B**) LOR resist was spin coated at 2000 rpm for 45 s on to the silicon substrate and a soft bake was performed at 150 °C for 150 s. (**C**) AZ701 resist was spin coated at 3000 rpm for 45 s on top of the LOR resist layer and a soft bake was performed at 110 °C for 75 s. (**D**) A Quintel Ultra i-line Series machine was used to pattern the resist layers using UV light with λ = 365 nm at an intensity of 6 mW/cm^2^ for 20 s. Pattern development was performed via immersion in a 2.3% tetramethylammonium hydroxide/97.7% water bath (**E**). The patterned silicon substrate was transferred to a CCS CA-40 E-beam Evaporator to deposit 350 nm of Ti. (**F**) Lastly, the wafer was immersed in NMP (1-methyl-2-pyrrolidone) to dissolve the AZ and LOR layers and effectively lift-off Ti from undesired regions of the substrate (Figure 1F). (**G**) Conceptual schematic of the cross section of an experimental device. (**H**) Experimental device and details of electrode dimensions and predefined regions of interest surrounding a single semicircular electrode.

Different electrode designs were considered for this work. The traditional interdigitated fingers, where targets are trapped along the entire length of the electrode, were first considered, but were quickly discarded since such design would not afford for punctual locations to enrich the parasite and facilitate their observation at specific and pre-determined spots. We then considered triangular electrodes because they are known to create punctual and strong electric field gradients [25], but abandoned this geometry for fear that the field strength at the sharp vertices of the electrodes would damage the integrity of the parasites (see Figure S1 in supplementary information). We finally settled on semi-circular electrodes with the rationale that semi-circles would offer weaker field gradients than the triangular electrodes but maintain the ability to safely enrich the parasites at specified locations. To this end, the semi-circles were arbitrarily positioned 3000 µm apart center-to-center to allow for separate, well-defined locations for potential enrichment (Figure 1H). 

The titanium electrodes were fabricated on a silicon oxide surface through a lift-off process, as shown in Figure 1A–F and detailed next. Silicon wafers (100 mm) featuring a 500 nm-thick thermal oxide (Noel Technologies, Inc. Campbell, CA, USA) were first cleaned in oxygen plasma (20 µTorr) for 15 s. A layer of LOR resist (Microchem, Newton, MA, USA) was spin coated for 45 s at 2000 rpm on the silicon substrate and baked at 150 °C on a hotplate for 150 s (Figure 1B) (Brewer Sciences Cee Spin Coater and integrated hotplate). A layer of AZ701 photoresist was then spin coated for 45 s at 3000 rpm on top of the LOR, baked at 110 °C for 75 s and exposed to a light with λ = 365 nm and an intensity of 6 mW/cm^2^ for 20 s to generate a pattern (Quintel Ultra i-line Series). Post exposure bake was done on the hotplate for 60 s at 110 °C. The exposed AZ and LOR layers were then immersed in a 2.3% tetramethylammonium hydroxide/97.7% water bath to develop the AZ layer and underetch the LOR (Figure 1D). The immersion time was manually adjusted to around 2 min following visual inspection until obtaining an underetch of the AZ layer of about 2 µm. After rinsing and drying, the patterned silicon substrate was transferred to a metal evaporator to deposit 350 nm of Ti (CCS CA-40 E-beam Evaporator). After the deposition process, the arrangement was immersed in remover NMP (1-methyl-2-pyrrolidone) to dissolve the AZ and LOR layers and effectively lift-off Ti from undesired regions of the substrate (Figure 1F). 

To ready the device for experimentation, a microfluidic chamber was created manually by cutting a rectangular shape from a paraffin film (PARAFILM^®^ M) and positioning the film around the Ti electrode array. For each experiment, 10 µL of cell sample was introduced into the chamber via micropipetting and a glass slide was used to cover the sample. Pressure was manually applied to the glass slide to compress the paraffin film and ensure the chamber was sealed. The cross section of an experimental device at the DEP region is shown in Figure 1G.

### 2.2. Sample Preparation and Viability Study

Samples of procyclic form (PCF) *T. brucei brucei* were obtained from the Morris Laboratory at the Eukaryotic Pathogens Innovation Center (EPIC) in Clemson University. *T. brucei* were cultured at 29 °C in 5% CO_2_ in SDM-79 media with a target density between 5 × 10^5^ and 1 × 10^7^ cell/mL [26]. *T. brucei* are known to display a worm like morphology reaching 20–40 µm in length by 1–3 µm in width [27]. Their normal behavior in culture media is that of a well dispersed population of individual parasites that are highly motile. 

The procedure to prepare experimental samples was optimized as follows. In order to induce the trapping of parasites in specific locations using positive DEP, the parasite must feature higher electrical polarization than its surrounding medium at a given frequency. Practically speaking and at the frequencies used in this work, the electrical conductivity of the medium must thus be as low as possible while also supporting the viability of the parasite during experiments. The requirement for low media conductivity is because complex permittivity $\varepsilon^{*}$ of the parasite $\varepsilon_{p}^{*}$ and media $\varepsilon_{m}^{*}$ are described by the same general equation

(1) $$\varepsilon_{p,m}^{*}=\varepsilon_{p,m}-j\frac{\sigma_{p,m}}{\omega }$$

where j is the imaginary unit vector; ω is the angular frequency of the applied electric field; and $\varepsilon_{p,m}$ and $\sigma_{p,m}$ are the permittivity and conductivity of the parasite or suspending media respectively. Analysis of Equation (1) illustrates how the complex permittivity is directly related to the electrical conductivity, and that to induce a positive DEP force on the parasite the conductivity of the media, $\sigma_{m}$, must be lower than that of the parasite, $\sigma_{p}$.

To this end, viability studies of the parasite in media with decreasing values of electrical conductivity were then conducted as follows. A sugar solution (9% sucrose, 0.5% dextrose and 0.3% bovine serum albumin by weight) widely used as buffer for DEP experiments was used as a base and its electrical conductivity adjusted to specific values using phosphate buffered saline (PBS). Conductivity values tested were 120 µS/cm (conductivity of the DEP experimental media), 408 µS/cm and 504 µS/cm (OAKTON PC700 conductivity meter). The control experiment was in growth media, SDM-79. In all cases, including the control, the parasites were washed and re-suspended three times into the designated media using centrifugation (Hermle Z200A). The different samples containing the parasites were then individually placed in 35 mm sterile petri dishes. A randomized area of each sample was observed and recorded for 30 min. This time was chosen as the maximum time for our viability study because our experiments were designed to be time efficient such that the results could compete with current clinical practices. Total clump area was measured to assess the health of the culture, since clumping is a common result of cell lysis due to factors such as environmental stress and overgrowth. An optimized media for DEP experiments was assumed to be that with the smallest electrical conductivity and lowest density of parasite clumps.

### 2.3. Computational Modeling

ANSYS Electronics Desktop running on a DELL XPS 15 with an intel Core i7-6700HQ CPU and 16 GB of RAM was utilized to model the distribution of the electric field (***E***) and the square of the electric field gradient (▽ ***E***^2^) using the built-in Maxwell 2D electrostatic solvers. The magnitude and spatial distribution of the electric field were modeled for four different values of polarization voltage in the range 5–20 V_pp_ towards selecting a voltage that would enable DEP forces but prevent electrical lysis of the parasite. Upon selecting such voltage, the corresponding ▽ ***E***^2^ was modeled to estimate the strength of the DEP force throughout the device and predict the regions we expected to lead to parasite enrichment.

### 2.4. Protocol for the Dielectrophoretic and Regional Enrichment Characterization of the Parasites

The experimental protocol did not feature any flow and all experiments were done in stationary flow conditions. Each experiment featured the following stages: (1) 10 µL of the experimental sample was injected into the microfluidic chamber of the device using a micropipette; (2) the chamber was sealed, and the sample allowed time to stabilize; (3) a video recording (Andor Zyla Camera coupled to a LV100 Nikon Eclipse Microscope) of the experiment was started; (4) after 10 s the titanium electrode array was polarized using an AC signal of specific frequency (100 kHz–20 MHz) and magnitude (5 V_pp_) using a BK Precision 4040B voltage generator. The response of the parasites to the polarized electrodes was recorded for 110 s. 

All 120 s-long videos were analyzed using ImageJ software [28]. The field of view for all videos recorded included 8 electrodes (Figure 1H). Only individual *T. brucei* that were on the same plane as the electrode, i.e., those that were in focus, were included in the analyses. 

## 3. Analytical Methods

### 3.1. Dielectrophoretic Characterization of the Parasites

The DEP response of the parasites was characterized based on the percentage of the parasites that were attracted to any part of the electrodes at the time mark of 50 s. The percentage of attracted parasites was calculated using Equation (2) by comparing the total number of parasites visible in the field of view at 50 s, or C_T,t = 50_, to the number of parasites that were attached (assessed visually by their characteristic perpendicular alignment to the electrode edge) to any of the 8 monitored electrodes, or C_A,t = 50_.

[C_A,t = 50_/C_T,t = 50_] × 100, (2)

This analysis was done for three videos per each of the 6 frequencies investigated in the range of 0.1–20 MHz. A higher percentage of attached cells was assumed to indicate a stronger positive DEP response induced on the parasites. 

### 3.2. Assessment of the Regional Enrichment of the Parasites

The ability to enrich the *T. brucei* parasites in specific locations was measured by monitoring the number of them over time in 4 unique and pre-defined regions of interest for each electrode, as illustrated in Figure 1H. Parasite enrichment was measured for each region and reported as a percentage increase or decrease in parasites from time t = 0 to t = 50 s. The average regional enrichment was plotted for each of the 4 defined regions to determine their enrichment potential. The percent enrichment for each region was calculated as a percentage change in the number of parasites from time t = 0 s to time t = 50 s using Equation (3).

[(C_RT,t = 50_ − C_RT,t = 0_)/C_RT,t = 0_] × 100, (3)

where C_RT,t = 50_ is the regional total count of parasites in at 50 s and C_RT,t = 0_ is the regional total count of parasites at 0 s for the same region. Only single, living parasites that were on the same focus plane of the electrode were considered. This consideration was practical and towards an eventual tool to facilitate direct observation of the parasites in specific locations. In this study positive values in enrichment percentage indicate a tendency for parasites to migrate towards the region of interest, while negative values indicate the opposite.

## 4. Results and Discussion

### 4.1. Optimizing the Experimental DEP Media for T. brucei

**Figure 2:**
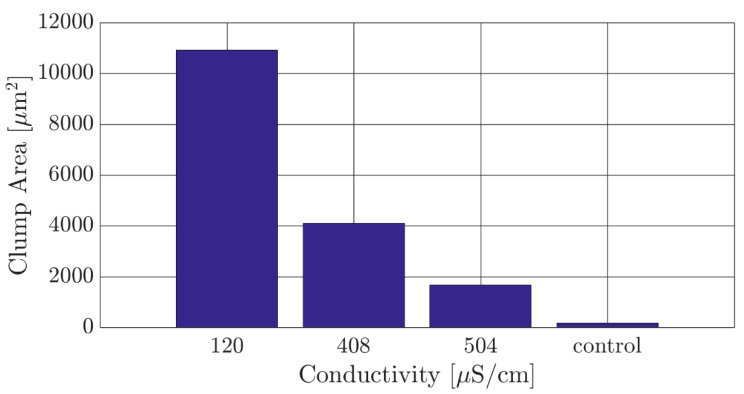
The total clump area of *Trypanosoma brucei* measured in different samples featuring a standard dielectrophoresis (DEP) experimental medium (120 µS/cm) and in experimental media with increasing electrical conductivity. The control was a sample of *T. brucei* in their standard culture media. An experimental medium with electrical conductivity of 504 µS/cm was chosen as a compromise between maintaining a suspension of individual parasites and a conductivity value that can lead to a strong positive DEP response.

As previously noted, the parasites are highly motile and maintain a single parasite dispersion when immersed in culture media. Since single parasite dispersion is necessary for adequate characterization of their DEP response, the ideal experimental DEP media would feature low electrical conductivity and lead to the least clump formation. Results of total clump area in the culture depending on the electrical conductivity of the media are shown in Figure 2 after an immersion time of 30 min. A clump of parasites was defined as three or more parasite sharing a single junction, and the 2D surface area of each clump was measured in ImageJ software [28]. The reported clump area is the summation of all clumps in the measured area. It is clearly observed that the total clump area decreased as the conductivity of the buffer media increased. The 504 µS/cm buffer was selected for our DEP experiments due to offering the best compromise between maintaining a low clump area and a conductivity that is low enough to potentially induce a positive DEP response within the parasites.

### 4.2. Optimizing Polarization Voltage for DEP Experiments

**Figure 3:**
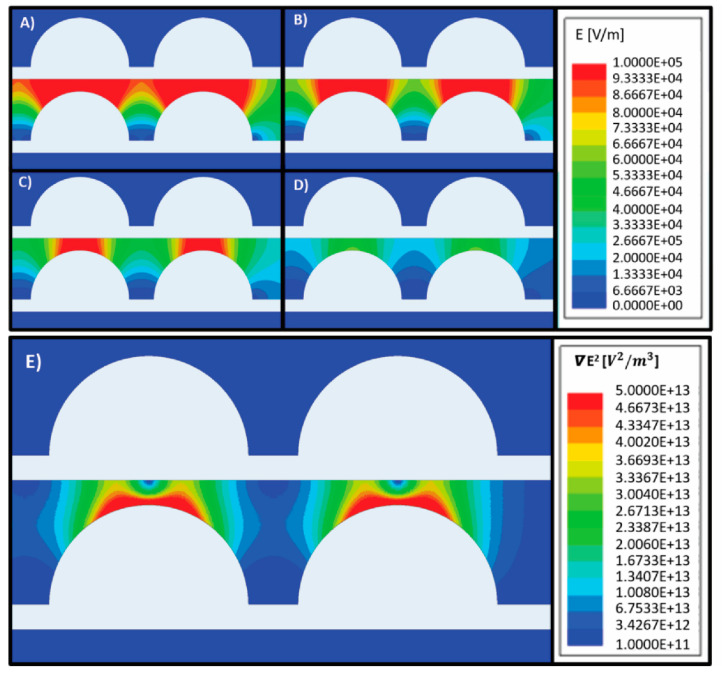
Modeling of the electric field ***E*** for an array of titanium electrodes (white geometries) polarized using different voltages: (**A**) 20 V_pp_ (**B**) 15 V_pp_ (**C**) 10 V_pp_ and (**D**) 5 V_pp_. The modeled media around electrodes was water with an electrical conductivity of 504 µS/cm. As expected, the magnitude of the electric field increases proportionally to the magnitude of the polarizing voltage. A magnitude of ***E*** < 10^5^ V/m is desired to minimize the risk of electrically lysing the parasites. (**E**) Modeling of ▽ ***E***^2^ in an array of titanium electrodes (white geometries) polarized using 5 V_pp_. The modeled media around electrodes was water with an electrical conductivity of 504 µS/cm. If the parasites experience a positive DEP force, they are expected to migrate to the regions of highest ▽ ***E***^2^, shown as orange–red in the figure.

Figure 3 depicts the distribution of ***E*** in the microfluidic device at different polarization voltages in the range 5–20 V_pp_. As expected, the magnitude of ***E*** is directly proportional to the polarization voltage (Figure 3A–D). In this work we targeted an electric field magnitude less than $10^{5}$ V/m throughout the entire device in order to maintain cell viability, by following the work by Glasser et al., who observed that electric fields of this magnitude showed minimal effects on cell viability during short term exposure to strong ac fields in the frequency range of our experiments [29]. Hence, we performed experiments using a polarization voltage of 5 V_pp_. In this case, the magnitude of ***E*** throughout the device would be expected to be <7 × 10^4^ V/m. 

Figure 3E illustrates the modeled distribution of ▽ ***E***^2^ in the microfluidic device when electrodes are polarized using 5 V_pp._ When assuming induction of a positive DEP force on the parasites, the parasites would migrate towards regions with the highest ▽ ***E***^2^, or the orange–red regions in the figure. Based on the results by Menachery et al., a magnitude of ▽ ***E***^2^ above $10^{13}$ $V^{2}/m^{3}$ would be enough to induce movement on the parasites [18]. Hence, from this computational model we would expect that parasites under the effect of a DEP force would migrate to the leading edges of the semicircular electrodes, which are included in regions 3 and 4 (see Figure 1H). 

### 4.3. Characterizing the Dielectrophoretic Response of T. brucei

**Figure 4:**
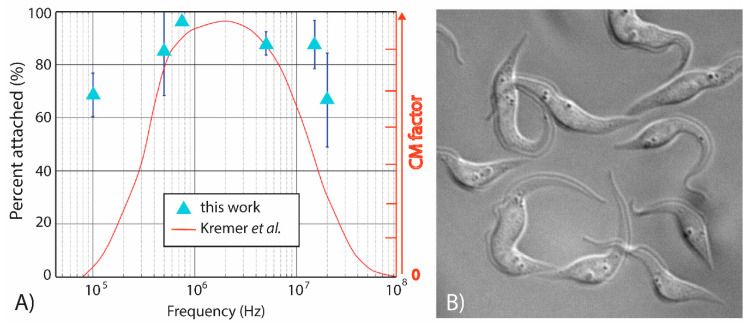
(**A**) Characterization of the DEP response of *T. brucei* across a broad frequency range, 100 kHz to 20 MHz. Dark blue bars represent the standard deviation between experiments (*n* = 3). (**B**) *T. brucei* cultured at 29 °C in 5% CO_2_ in SDM-79 media with a target density between 5 × 10^5^ and 1 × 10^7^ cell/mL. Courtesy of Christina Wilkinson and James Morris.

Electrical charges naturally exist within the cell structure and these can become redistributed and aligned upon exposure of the cell to an electric field. This leads to cell polarization, and the inductance of an electric dipole and motion of the cell due to DEP [30]. The electrical double-layer that develops at the interface between the cell outer envelope and the suspending electrolyte will yield a membrane capacitance that depends on the cell size, shape and composition of said outer envelope. Such capacitance will dominate the DEP response of the cell at low frequency values of the applied electric field, i.e., the leftmost region of the DEP curve in Figure 4. In addition to this interface, the organelles and entities within a cell will yield their own dipole depending on their unique structure and function. At higher frequencies of the applied electric field, the cell’s DEP response becomes a function of the electrical properties of the interfaces inside the cell, such as those originating from different organelles in the cytoplasm and their volume relative to that of the cytoplasm [30,31]. 

Figure 4 depicts the DEP response of *T. brucei* to an applied voltage excitation of 5 V_pp_ at six different frequencies in the range 100 kHz–20 MHz. The light-blue triangles in Figure 4A represent the average (*n* = 3) attachment percentage of *T. brucei* to the electrode at a given frequency. A theoretical DEP response of *T. brucei*, shown as the smooth red curve, was previously reported by Kremer et al. [17] and overlaid over our results for comparison and discussed next. It can be clearly observed that the percentage of attachment in the experiments performed here was at least 50% and that a polarizing frequency of 750 kHz leads to the strongest positive DEP force and highest percent attachment, thereby providing the most potential to rapidly enrich the parasites in a specific location. These results also indicate that *T. brucei* shows a strong positive DEP response across the entire frequency range tested—a fact that is partially confirmed by Kremer et al. [17]. While experimental and theoretical results highly overlap at frequencies above 500 kHz, the theoretical DEP trapping seems to sharply decrease at frequencies below 400 kHz. Disparities between our experiments and Kremer et al. at these lower frequencies can be accredited to the shape simplifications of the parasite. Their assumptions simplify the parasite into a prolate elliptical shape, composed of two concentric shells, a membrane and cytoplasm. As illustrated in Figure 4B and reported by other authors, the parasites feature a worm-like morphology vastly more complex in shape and internal structure [32] than what was assumed by Kremer and co-authors [17]. Large organelles present inside the parasite, such as the nucleus and the kinetoplast, each would have their own dielectric properties, and together with their spatial distribution, would contribute to the overall dielectric response of the parasite. Other organelles within the parasite are also likely to have an effect on its DEP response, especially at high polarizing frequencies. Hence, the broadening of our experimental curve when compared to Kremer et al. is likely due to the slender shape and size of the parasite at frequencies in the kHz range and the contributions of the different organelles at frequencies beyond 10^7^ Hz. A detailed study on the impact of organelles on the DEP response of *T. brucei* is out of the scope of this paper, which focuses on determining the conditions that will lead to rapid and strong enrichment of the parasite in specific locations. Envisioned future work includes such a detailed study and the effects of parasite motility and age on the DEP behavior of *T. brucei*.

### 4.4. Determining the Region that Yields the Highest Enrichment of T. brucei

**Figure 5:**
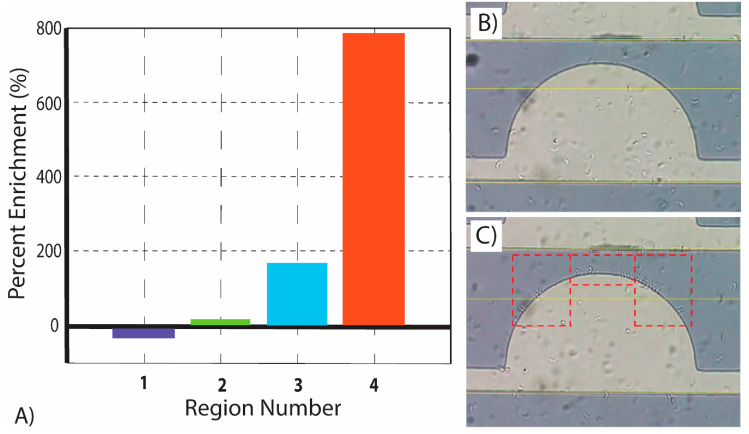
(**A**) Regional enrichment study of parasites from time t = 0 to t = 50 s for four predefined regions of interest shown in Figure 1H. All enrichment experiments were performed at a frequency of 750 kHz, as such frequency yields the strongest positive DEP response of *T. brucei* under the conditions studied in this work. (**B**) Single electrode at t = 0 illustrating low attachment of parasites to electrode edges. (**C**) Single electrode at t = 50 illustrating high attachment of parasites to electrode edges, particularly in regions 3 and 4 (dashed rectangles).

Figure 5 depicts the results of the enrichment study, where the enrichment of parasites is expressed as a percentage of increase or decrease in parasites from time t = 0 to t = 50 s within each of the four pre-defined regions of interest. Region 4 yields close to 800% enrichment while region 1 is actually depleted of parasites (negative enrichment). Importantly, the percentage of enrichment reported is a combination of both enrichment due to the sideways migration of parasites from one region to another, and enrichment from parasites migrating from the bulk of the sample into the observation plane. 

During analysis it was confirmed that the parasites tended to concentrate in regions of higher electric field gradient. Experiments showed that parasites tended to concentrate in region 4, a square area of dimensions 40 µm × 85 µm, at a rate of 780%, higher than any other region. In fact, the second highest rate was region 3, which increased the concentration of parasites by 163% over the same time span. Region 1 resulted in an average decrease in the concentration of parasites by −29%, while region 2 saw the parasite concentration increase by only 12%. The computational model for ▽ ***E***^2^ presented here further validates these results due to the fact that the regions with the highest enrichment correlated to the regions of the electrode with the highest ▽ ***E***^2^. These results indicate a significant potential for the use of DEP to position and enrich the concentration of *T. brucei* in specific locations. Particularly, our microfluidic chamber facilitated the ability to increase parasite concentration in region 4, a square area of dimensions of 40 µm × 85 µm, by 780% in 50 s.

## 5. Concluding Remarks

In this work we contribute a study of the conditions that led to the enrichment of *T. brucei* in specific locations using DEP. A frequency of 750 kHz at a polarizing voltage of 5 V_pp_ induced the strongest positive DEP response from the *T. brucei* parasites. This frequency was subsequently utilized to position and enrich *T. brucei* within a square planar region 40 × 85 µm. The positioning proved to be highly efficient, resulting in a 780% enrichment of parasites in less than a minute.

Early diagnosis of the presence of *T. brucei* at the first stage of infection can have a significant impact on patient outcome by enabling timely and adequate treatment before the disease moves into the second stage, which causes neuropsychiatric manifestations, such as sleep disorders, derangement and eventually death. Upon positive results from a CATT, assessing the presence of *T. brucei* in locations determined *a priori* can facilitate their detection and thus lead to an easy-to-use and robust assay. The use of arrays of semicircular titanium electrodes to enrich parasites in desired locations using DEP shows high potential to achieve this end. However, further work is needed to characterize the specificity of DEP in regard to enriching *T. brucei* in a practical scenario. More specifically, the ability to use DEP to isolate parasites from WBCs and other species, i.e., microorganisms and parasites, might be present in a buffy coat and/or plasma portions of a centrifuged blood sample.

## Supplementary Materials

The following are available online at https://www.mdpi.com/2072-666X/11/6/625/s1, Figure S1: Modeling of the electric field ***E***for an array of triangular titanium electrodes (white geometries) with the same footprint than the semi-circular electrodes presented in the main text.
