# Supplementary material for: Highly Localized Enrichment of Trypanosoma brucei Parasites Using Dielectrophoresis
Source: Micromachines (Basel). 2020 Jun 26;11(6):625. doi: 10.3390/mi11060625 (PMC7344920; doi:10.3390/mi11060625)
Supplement: Supplementary file 1 [file micromachines-11-00625-s001.pdf]

Supplementary Materials: Highly Localized Enrichment of *Trypanosoma brucei* Parasites Using Dielectrophoresis

Devin Keck, Callie Stuart, Josie Duncan, Emily Gullette and Rodrigo Martinez-Duarte

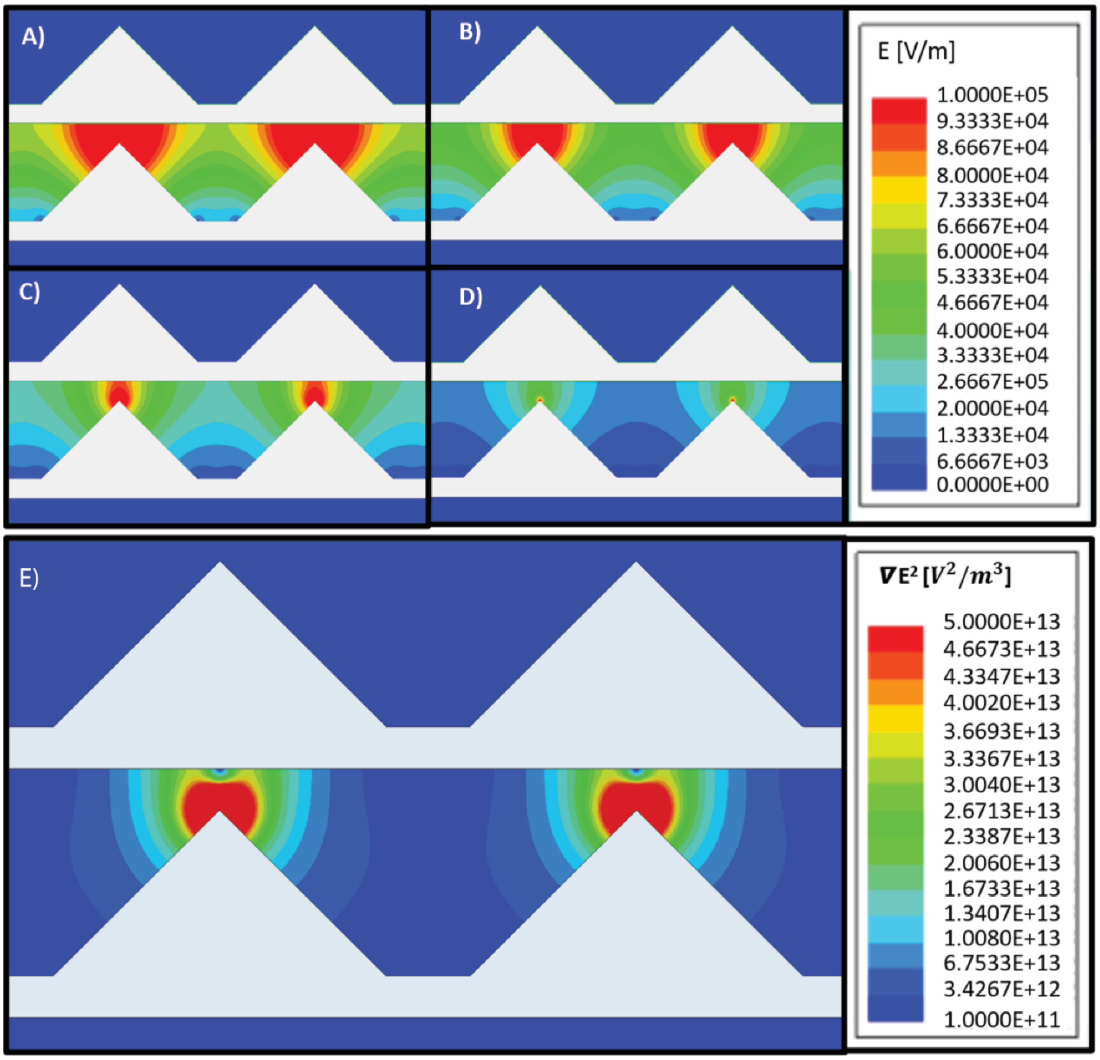

Figure S1. Modeling of the electric field $\boldsymbol{E}$ for an array of triangular titanium electrodes (white geometries) with the same footprint than the semi-circular electrodes presented in the main text. Electrodes are modeled to be polarized using different voltages: (**A**) 20 V_{PP} (**B**) 15 V_{PP} (**C**) 10 V_{PP} and (**D**) 5 $V_{PP.}$ The modeled media around electrodes was water with an electrical conductivity of 504 µS/cm. (**E**) Modeling of $\boldsymbol{\nabla} E^2$ in the array of triangular titanium electrodes (white geometries) when polarized using 5 V_{PP}. The modeled media around electrodes was water with an electrical conductivity of 504 µS/cm.
